# Computer-Aided Intracranial EEG Signal Identification Method Based on a Multi-Branch Deep Learning Fusion Model and Clinical Validation

**DOI:** 10.3390/brainsci11050615

**Published:** 2021-05-11

**Authors:** Yiping Wang, Yang Dai, Zimo Liu, Jinjie Guo, Gongpeng Cao, Mowei Ouyang, Da Liu, Yongzhi Shan, Guixia Kang, Guoguang Zhao

**Affiliations:** 1 Key Laboratory of Universal Wireless Communications, Ministry of Education, Beijing University of Posts and Telecommunications, No. 10 Xitucheng Road, Haidian District, Beijing 100876, China; ypwang@bupt.edu.cn (Y.W.); liuzimo@bupt.edu.cn (Z.L.); guojinjie2@bupt.edu.cn (J.G.); gpcao@bupt.edu.cn (G.C.); baytest@bupt.edu.cn (M.O.); 2 Department of Neurosurgery, Xuan Wu Hospital, Capital Medical University, No. 45 Changchun Street, Xicheng District, Beijing 100053, China; yangdai@mail.ccmu.edu.cn (Y.D.); shanyongzhi@xwhosp.org (Y.S.); 3 Robotics Institute, School of Mechanical Engineering & Automation, BeiHang University, No. 37 Xueyuan Road, Haidian District, Beijing 100191, China; drliuda@buaa.edu.cn; 4 Wuxi BUPT Sensory Technology and Industry Institute Co. Ltd., Wuxi 214001, China

**Keywords:** intracranial EEG (iEEG), SEEG, epileptogenic signals identification, multi-branch deep learning fusion

## Abstract

Surgical intervention or the control of drug-refractory epilepsy requires accurate analysis of invasive inspection intracranial EEG (iEEG) data. A multi-branch deep learning fusion model is proposed to identify epileptogenic signals from the epileptogenic area of the brain. The classical approach extracts multi-domain signal wave features to construct a time-series feature sequence and then abstracts it through the bi-directional long short-term memory attention machine (Bi-LSTM-AM) classifier. The deep learning approach uses raw time-series signals to build a one-dimensional convolutional neural network (1D-CNN) to achieve end-to-end deep feature extraction and signal detection. These two branches are integrated to obtain deep fusion features and results. Resampling is employed to split the imbalanced epileptogenic and non-epileptogenic samples into balanced subsets for clinical validation. The model is validated over two publicly available benchmark iEEG databases to verify its effectiveness on a private, large-scale, clinical stereo EEG database. The model achieves high sensitivity (97.78%), accuracy (97.60%), and specificity (97.42%) on the Bern–Barcelona database, surpassing the performance of existing state-of-the-art techniques. It is then demonstrated on a clinical dataset with an average intra-subject accuracy of 92.53% and cross-subject accuracy of 88.03%. The results suggest that the proposed method is a valuable and extremely robust approach to help researchers and clinicians develop an automated method to identify the source of iEEG signals.

## 1. Introduction

Intractable epilepsy is now recognized as a disease with significant morbidity and mortality, resulting in severe threats to patients’ physical and mental health [1,2,3]. With the aim of the precise administration of the disease to achieve a better outcome, invasive inspection techniques are essential for the articulation of specific epileptogenic and non-epileptogenic signals [4]. Different intracranial EEG (iEEG) invasive inspection techniques, such as electrocorticography (ECoG) and stereo-electroencephalography (SEEG), have been established to locate epileptogenic focus [5]. iEEG signals provide anatomically precise information about the selective engagement of neuronal populations at the millimeter scale and the temporal dynamics of their engagement at the millisecond scale, and they play a dominant role in the discovery and detection of particular zones in the impacted brain [6]. In clinical applications, ECoG must be recorded after the brain cortex is exposed, and the recording can last for only minutes during the interictal period. SEEG is considered the “gold standard” method to evaluate the epileptogenic zones for electrodes implanted in the deep brain and can record interictal and ictal epileptic discharges for days before deciding the extent of resection [6,7,8,9]. Our team at the China International Neuroscience Institute (China-INI) of XuanWu Hospital has also confirmed the value of SEEG in the assessment and guidance of thermocoagulation of epileptogenic foci [10].

However, visually determining abnormal discharges in a patient’s iEEG recording is a tedious task for clinicians [11,12]. To improve this situation, an automatic computer-identification method is necessary to provide the right support for clinical experts. Its advantages can be reflected in the following three aspects. First, computer-aided epileptogenic signal identification is efficient, time saving and can assist low- and middle-income countries and inexperienced doctors to a greater extent. Second, features that cannot be easily detectable by human visual inspection can be identified by the computer localization method. Third, the accuracy rate can be kept stable and is not affected by subjective factors during computer operation.

Computer-aided epileptogenic signal identification is carried out in two stages. In the first stage, the main purpose is to extract significant difference features to distinguish the epileptogenic and non-epileptogenic signals—that is, extract highly sensitive digital features or advanced abstract hidden layer features of abnormal patterns from the raw iEEG signal. In the second stage, the different features of the previous step are sent to the classifier for signal classification, which completes the identification of epileptic signals. However, the end-to-end architecture of deep learning (DL) mostly integrates the first and second stages to complete the task.

The key content of the first stage of epileptogenic signal identification is to extract effective features to fully represent the signal [13]. Multiple-domain feature extraction, such as the time, frequency or combined time–frequency domain extraction [14,15,16], and nonstationary feature domain analyses [17,18,19] have been used to perform the epileptic seizure detection task [20] and epileptogenic signal identification task [21]. Li et al. [14] converted the signals into high-resolution time–frequency diagrams for feature extraction, and then discriminative features were obtained according to 5 sub-bands of clinical interest. In addition, our team [15] used multifractal analysis methods based on the generalized Hurst exponent, Hurst exponent, fluctuation index, and mean and standard deviation to extract important features and obtained satisfactory results. However, two main deficiencies are involved in these methods. First, nonlinear characteristics of EEG cannot be well represented. Second, when processing iEEG data with a high sampling rate, the amount of calculation increases, resulting in a decrease in the accuracy of these methods. Furthermore, the nonstationary feature domain has been proved effective in recent research. In [17], iEEG signals were decomposed according to FAWT, and the log energy entropy and fuzzy distribution entropy of 15 sub-bands were computed. Machine learning algorithms were then used to verify the effectiveness of the selected features. In a follow-up study, Rahman et al. [18] utilized features obtained from variational mode decomposition and the discrete wavelet transform (DWT) domain, as well as improved composite multiscale dispersion entropy, fuzzy entropy and other features. These entropy and nonstationary feature domains can represent higher time–frequency resolution and better reflect the nonlinear dynamics of the brain captured in the iEEG signal. Although these methods have obtained encouraging results, they also suffer from decreases in the efficiency of feature weight evaluation and show moderate accuracy when machine-learning methods face large numbers of multi-domains and multi-features.

DL has been proved to be very efficient in many complex biomedical tasks [22,23,24,25], especially in EEG signal aspects. A long short-term memory (LSTM) network works well with time-series information due to its structural dependency [26,27], and the attention mechanism (AM) [28,29] has the ability to focus on the abnormal signals of EEG. In addition, convolutional neural networks (CNNs) can detect and extract relevant features automatically [30]. The studies demonstrate that the DL solution can not only further abstract traditional features but also extract weak features that cannot be found visually on the original signal. However, it also suffers from complicated model tuning and poor interpretability of the deep structure when facing high-dimensional and high-resolution data.

In the second stage, how to integrate different features to build a useful and accurate classification model is an important breakthrough to achieve signal identification. In the traditional method, the manually extracted features are sent to a machine-learning classifier to perform feature selection to realize classification [15,31]. With the extraction of multi-dimensional and multi-perspective features and the development of DL, many studies have sent the obtained full-channel features into the DL model to complete signal classification while abstracting high-level features [26,27]. Furthermore, DL end-to-end architecture, complete feature extraction, and signal classification have been implemented at the same time [30]. The above research provides theoretical and method support for realizing accurate and robust epileptic signal detection. However, specific problems, such as the specific identification of iEEG signals, remain to be studied.

To overcome the above shortcomings, a multi-branch DL fusion model is proposed for epileptic and non-epileptic signal identification. The main contribution of the proposed multi-branch DL fusion model is that it considers not only the signal wave features but also deep high-order features. Both branches use the DL model (Bi-LSTM-AM and 1D-CNN) as the classifier to high-level abstract the epileptogenic signal features based on the time-series feature sequence and raw time-series signal and then accurately identify two-class signals. The proposed method is extremely robust while ensuring accuracy.

Another unique contribution is that we have not only achieved state-of-the-art performance in the databases of two public evaluation benchmarks but also better application in a real-world clinical database. Specifically, (a) resampling technology is employed to split the clinical database to overcome the limitation of extremely unbalanced data; (b) a useful recognition result is achieved in intra-subject and cross-subject validation; (c) finally, our study provides baseline methods and results for epileptogenic zone localization. We believe that this represents a breakthrough in data science and clinical epilepsy that will be very useful for precise preoperative positioning of pharmacoresistant epilepsy and the precise administration of the disease for a better seizure outcome.

## 2. Materials and Methods

### 2.1. Experimental Databases

**Figure 1:**
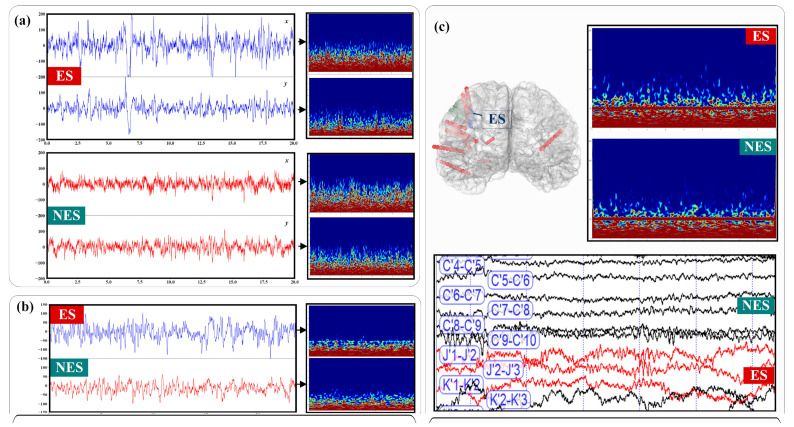
Three independent iEEG databases. (**a**) Epileptogenic signals (ES) and non-epileptogenic signals (NES) in public iEEG Bern–Barcelona database and its time–frequency diagram; (**b**) ES and NES in part of the small public iEEG Bonn University database and the time–frequency diagram. (**c**) The upper left is the patient’s brain image, in which the blue lead in the brain on the left is the epileptic lead, and the red lead in the brain on the left is the non-epileptic lead. Below are clinical signals in the SEEG database, and in the upper right is the time–frequency diagram of ES/NES.

Three independent iEEG databases are employed to evaluate the proposed epileptogenic signal identification approach, including two publicly available benchmark iEEG databases and one clinical SEEG database. The first two public databases are used to build the proposed model and provide public evaluation criteria, and the third clinical database is used to verify the reliability, accuracy and robustness of the approach in real clinical application scenarios. Figure 1 shows an example of the three independent iEEG database signals.

#### 2.1.1. Public iEEG Bern–Barcelona Database

These public iEEG data were from the Bern–Barcelona database [21], which included two categories of iEEG recordings from five epilepsy patients who suffered from drug-resistant, long-standing temporal lobe epilepsy. This database is widely used in epilepsy research to solve extremely challenging tasks and to distinguish whether a signal originates from the brain epileptogenic zone (focal) or brain non-epileptogenic zone (non-focal)—that is, to determine whether it is an epileptogenic signal. The iEEG signals were recorded for 20 s at a sampling rate of 1024 Hz and then downsampled to 512 Hz. Each data segment contains 10,240 data points.

In the experiment of our study, the entire database is used to classify 3750 pairs of epileptogenic (focal) and 3750 pairs of non-epileptogenic (non-focal) iEEG signals to train and verify the proposed model.

#### 2.1.2. Part of Small Public iEEG Bonn University Database

This small iEEG dataset was obtained from the Bonn University database [20], which is widely used in seizure detection and consists of five subsets denoted as Z, O, N, F, and S, each of which contains 100 single-channel EEG segments of 23.6 s duration. All signals were recorded from the same 128-channel amplifier system with a sampling rate of 173.61 Hz.

Specifically, subsets Z and O are composed of scalp EEG segments acquired from healthy volunteers who were relaxed and awake with eyes closed and opened, respectively. Subset S comprises iEEG acquired from the epileptogenic zone during seizure activity.

However, considering that our study focuses on the interictal data of intracranial EEG, we selected the N and F subsets. The interictal iEEG segments in subset N were recorded from the nonepileptic area of the brain, and the interictal iEEG segments in subset F were recorded from the epileptogenic zone of the brain. Thus, discriminating focal iEEG from non-focal iEEG can be used to identify the epileptogenic signals of the brain.

#### 2.1.3. Private Clinical SEEG Database

The private clinical SEEG data were collected and maintained by the Department of Neurology Xuanwu Hospital in China.

For each patient, stereotactic EEG recordings were collected by a Nicolet 256-channel EEG detection system with a sampling rate of 2048 Hz. In the data collection of our study, electrodes were implanted in patients before undergoing resection or destructive surgery. No SEEG record was collected from the patients in the first two days after the implantation of the electrode. The original EEG recording of the patient was recorded from the third day after surgery. Thus, we selected the sleep data of the interictal period on the third and last days after surgery.

Our work selected the interictal period SEEG of the sleeping state of five patients with a single acquisition time of 2 h. We collected sleep period data based on the patient’s video recording, the period in which relative deep sleeping and turning over occurred was light. We cut the time series into contiguous segments of 10 s each, and each segment contained 20,480 data points. The entire dataset contained 64,890 SEEG signals in total. According to the diagnosis and experience of clinical experts, the signal with the lead label as the origin point was marked as an epileptogenic signal from the epileptogenic zone of the brain; otherwise, it was marked as non-epileptogenic signal data from the non-epileptogenic zone of the brain. The detailed information of the SEEG data for each patient is given in Table 1.

### 2.2. System Overview

**Figure 2:**
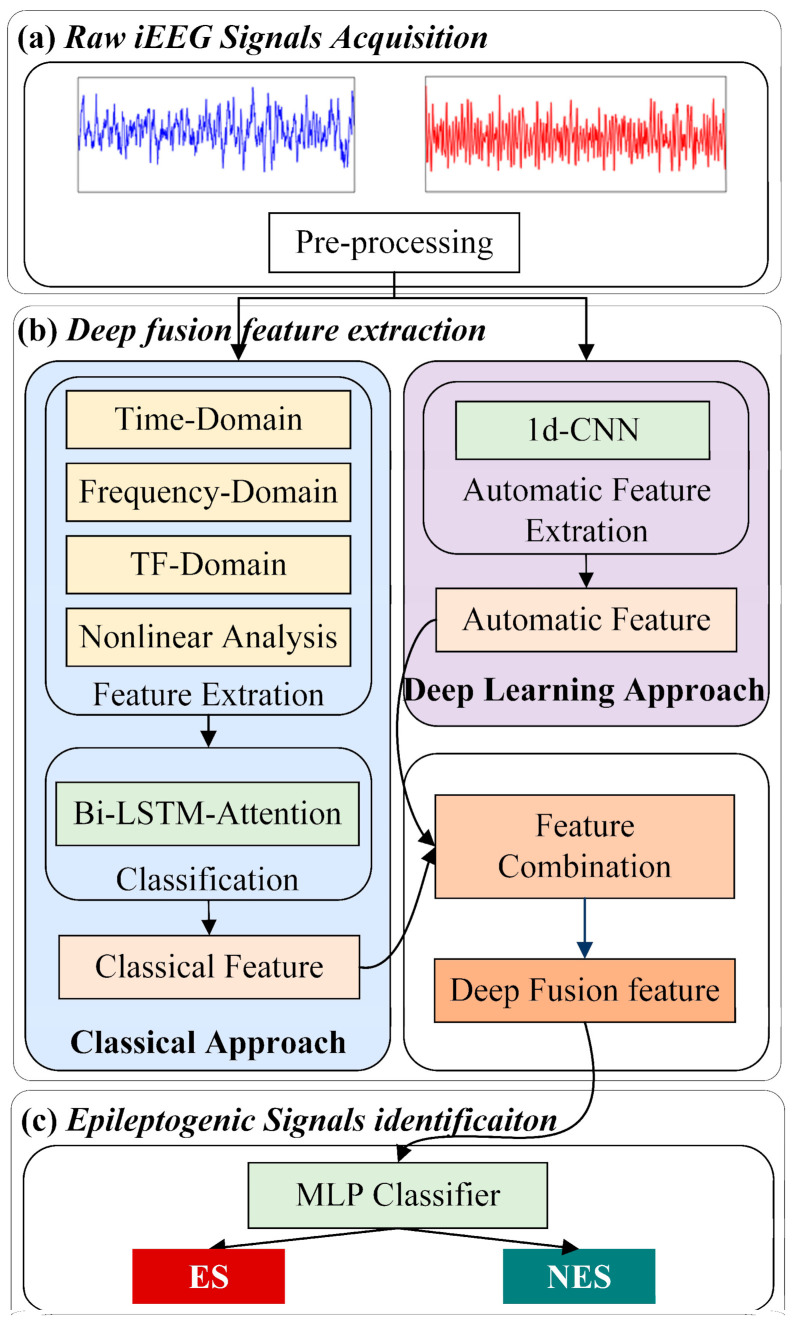
The framework of the proposed epileptogenic signal identification model. (**a**) ES (the blue one) and NES (the red one) were the input of this part; (**b**) combine the Classical Approach and Deep Learning Approach to get the Deep Fusion feature; (**c**) though an MLP Classifier to achieve ES identification.

The system overview of the proposed epileptogenic signal identification model is given in Figure 2. First, we obtained raw iEEG signals and performed preprocessing. Then, we sent them in parallel to the classical approach to obtain the classical feature and the end-to-end DL approach to obtain the automatic feature. The features from the two parts were combined to obtain deep fusion features. Finally, a multilayer perceptron (MLP) classifier was used to identify the result.

In this work, how to achieve a high accuracy rate and robustness in epileptic signal identification is the main problem. Moreover, how to obtain effective and stable feature extraction from raw iEEG signals is the most essential aspect. Thus, we not only performed the feature-based method to fully represent the epilepsy of the signal but also extracted the deep high-order feature of the signal through the end-to-end method based on DL.

### 2.3. iEEG Data Processing and Balance Treatments

#### 2.3.1. Data Processing for Public iEEG Bern–Barcelona Database

For the first Bern–Barcelona database, the same operation was first applied to the original two-channel iEEG signals. We used the Butterworth bandpass filter [32] to obtain the signal from 0.5 Hz to 80 Hz. Then, the signals were decomposed into sub-bands using DWT, and the wavelet coefficients obtained by the two channels were averaged. Among them, the frequency bands of the DWT decomposition coefficients A4, D4, D3, D2, and D1 corresponded to 0.5–5 Hz, 5–10 Hz, 10–20 Hz, 20–40 Hz and 40–80 Hz, respectively.

For the second small Bonn University database, for single-channel data, the above decomposition process was repeated.

#### 2.3.2. Data Balance for Private Clinical Database

Before data processing and feature extraction, a data-balance treatment for the private clinical database is proposed, based on the downsampling technique.

Suppose there are *M* leads from the majority class (non-epileptogenic leads) and *N* leads from the minority class (epileptogenic leads), where *M* > *N*. With the number of signals from non-epileptogenic leads as the upper limit, the signals from epileptogenic leads were resampled, that is, a sliding window was used for epileptogenic signals, and the resampling technique was used to equalize the number of two-class samples.

Specifically, in order to ensure that single SEEG acquisition and segmentation were complete with 10 s, we changed the size of the sliding window according to the ratio of *M* to *N*. The sliding window size is calculated as Equation (1):

(1) $$slid_{size}=CalIntegralMultiple\left(\frac{S}{M/N}\right)$$

where *CalIntetgralMultiple* is used to ensure that the start and endpoints of each signal are complete one or half-second data, and we set the sliding window size to multiples of 0.5.

Finally, we resampled using the sliding window, and the number of epileptogenic signals *n* is calculated as Equation (2) and non-epileptogenic signals *m* is calculated as Equation (3):

(2) $$n=\frac{dur}{S}$$

(3) $$m=fix\left(\frac{dur-S}{slid_{size}}\right)$$

where *dur* is the duration of a continuous signal segment, which may be half an hour or 2 h, depending on the data collection situation. *S* is the time of the segments, which is 10 s. *fix* is a function that rounds toward zero.

In summary, we used the above operations to overcome the imbalanced limitations of the clinical databases and form a balanced dataset, as shown in Table 1, to ensure that the model can fully learn the information of all channels and leads.

#### 2.3.3. Data Processing for Private Clinical Database

For the clinical SEEG dataset, to reduce the influence of unnecessary noise and artefacts on the location, our study removed the bad leads and the electrodes located in the functional area from the clinical data. Then, the signals were segmented into a Butterworth bandpass filter to obtain a signal from 0.5 Hz to 256 Hz. Other decomposition treatments were the same as in Section 2.3.1.

### 2.4. Epileptogenic Signal Identification by Classical Approach

In this approach, we performed feature extraction on the original signal after processing, divided it into 4 continuous segments, sent them to Bi-LSTM-AM to learn timing features, and then obtained the classical feature.

#### 2.4.1. Feature Extraction

iEEG is an intracranial nonstationary time series, and the analysis methods are usually divided into linear analysis and nonlinear analysis. The selected features of our study in the time–domain analysis mainly included 12 features. Frequency–domain analysis can intuitively reflect the distribution and changes of EEG signals in different frequency bands; thus, we mainly included 6 frequency domain features. In nonlinear feature analysis, we extracted different entropy features from empirical mode decomposition (EMD) and the wavelet coefficients of DWT. Detailed characteristics and formulas are shown in Table 2.

In the time–domain analysis, the selected features of our study mainly included the (a) mean; (b) variance; (c) coefficient of variation, which reflects the absolute value of the dispersion degree of iEEG data; (d) skewness; (e) kurtosis; (f) interquartile range; (g) activity of the Hjorth parameter, which reflects the variance of the average power of the iEEG signal; (h) mobility of the Hjorth parameter, which reflects the ratio of the root mean square of the signal’s slope to the root mean square of the signal’s amplitude, which is a parameter to estimate the mean frequency; (i) complexity of the Hjorth parameters, which reflects the signals’ ratio change and is used to estimate the bandwidth of iEEG signals; (j) zero crossing rate, which is low in places with high energy and high in places with low energy; (k) Hurst parameters; and (l) DFA fractal.

The frequency–domain features selected in our study mainly included the (a) sub-band power ratio; (b) power spectral density, which estimates the power spectrum of iEEG signals and transforms time–domain iEEG signals whose amplitude changes with time into the power spectrum of iEEG with frequency; (c) amplitude spectrum density; (d) spectral centroid; (e) spectral kurtosis; and (f) spectral entropy.

However, there is a limitation of time–domain and frequency–domain methods, and the precise frequency and time information involved in a specific time cannot be provided separately. To overcome these limitations, our study used EMD to adaptively analyze the main components of the signal and used DWT methods for nonlinear analysis, which can better reflect the distribution of signals.

The advantages of EMD methods do not need to be predetermined or forced to give a basis function when decomposing signals but depend on the characteristics of the signal itself and decomposes adaptively. We obtained 5 corresponding intrinsic mode function (IMF) components through EMD methods, and the fuzzy entropy value of the IMF component was calculated. Then, the sample quantile method was used to obtain the quantiles of the fuzzy entropy values of multiple IMF components. We returned a value with dimensions of 2*1 that contained the first and third quartile values.

Moreover, nonlinear feature extraction was performed based on DWT to obtain five-level multiresolution decomposition wavelet coefficients through a Butterworth bandpass filter, and each sub-band signal was characterized by 10 disparate entropies (as shown in Table 2). The extracted nonlinear features specifically included (a) Kraskov entropy, also called entropy estimation, which is used to compare the performance of the signal’s Shannon entropy; (b) Renyi entropy, which quantifies the diversity, uncertainty or randomness of iEEG signals; (c) permutation entropy, which is a dynamic mutation detection method, has an amplification effect on small changes in signals and can measure the complexity of iEEG signals; (d) sample entropy, which is used to measure the complexity of iEEG signals; (e) Shannon entropy, which reflects the uncertainty of iEEG signals; (f) energy; (g) SVD entropy (because of nonoverlapping bumps in the boxplot, it can be concluded that the true median was indeed different with 95% confidence—SVD entropy measures the richness of features, in a sense; (h) PFD; (i) KFD; and (j) HFD.

#### 2.4.2. Bi-LSTM-Attention Classifier with the Classical Approach

**Figure 3:**
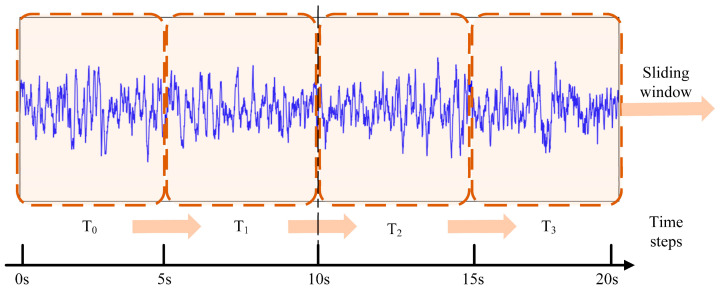
iEEG segments are illustrated (a 20 s LSTM sequence consists of 4 timesteps with no overlap between adjacent windows).

Figure 3 illustrates a sample iEEG recording and the four segments we divided with a sliding window, as $T_{0},T_{1},T_{2}$ and $T_{3}$. To present a personalized solution in epileptogenic signal identification, we enhanced the representation of Bi-LSTM with an attention machine (AM). The attention-based enhanced Bi-LSTM can facilitate the identification of the epileptogenic signals to learn specific features of different patients and tune our model to achieve accurate recognition of individual iEEG signals.

**Figure 4:**
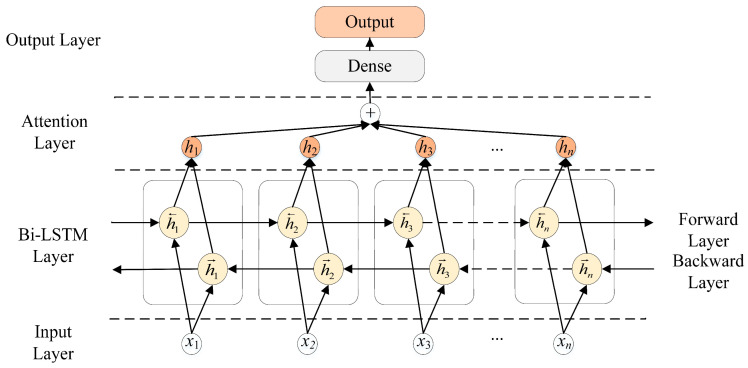
The structure of the Bi-LSTM network.

In the following subsection, we describe the architecture of a Bi-LSTM cell and the AM. Figure 4 depicts the overall structure.

(a) Bi-LSTM Network

LSTM is a recurrent neural network (RNN) that solves the gradient problem of disappearance and explosion by learning long-term and short-term dependencies. One LSTM processes the iEEG serials from left to right, and the other one processes them from right to left. At each time step t, a hidden forward layer with hidden unit function $\vec{h}$ is computed based on the previous hidden state $\vec{h_{t-1}}$ and the input at the current step $x_{t}$, and a hidden backward layer with hidden unit function $\overset{\leftarrow }{h}$ is computed based on the future hidden state $\overset{\leftarrow }{h_{t+1}}$ and the input at the current step $x_{t}$. The forward and backward context representations, generated by $\vec{h_{t}}$ and $\overset{\leftarrow }{h_{t}}$, respectively, are concatenated into a long vector. The combined outputs are the predictions of teacher-given target signals.

Bi-LSTM uses two LSTMs to learn each token of the iEEG serials based on both the past and the future context of the token (Figure 4).

(b) Attention Machine

An AM can improve the performance of Bi-LSTM by paying attention to the specific input feature with the most discriminative information. To capture the importance of each input segment, the AM is defined as Equations (4)–(6):

(4) $$u_{t}=\tanh\left(W_{w}e_{t}+b_{w}\right)$$

(5) $$h_{t}=\frac{exp\left(u_{t}^{T}u_{w}\right)}{\sum_{t}exp\left(u_{t}^{T}u_{w}\right)}$$

(6) $$v_{t}=\underset{t}{\sum }h_{t}\cdot e_{t}$$

where $v_{t}$ is the output of the attention layer, while $W_{w}$, $u_{w}$ and $b_{w}$ denote two trainable weights and the bias, respectively. Through multiplication of $e_{t}$ and $h_{t}$, it selects and extracts the temporal and spatial information from $e_{t}$ that contributes most significantly to the decoding tasks.

(c) Bi-LSTM-Attention

All 280 features from the 4 timesteps in each segment were fed to the first Bi-LSTM layer. The final Bi-LSTM layer was followed by an attention layer, which was, in turn, followed by a fully connected layer with a sigmoid activation function to predict the probability of each category.

### 2.5. Epileptogenic Signals Identification by DL Approach

**Figure 5:**
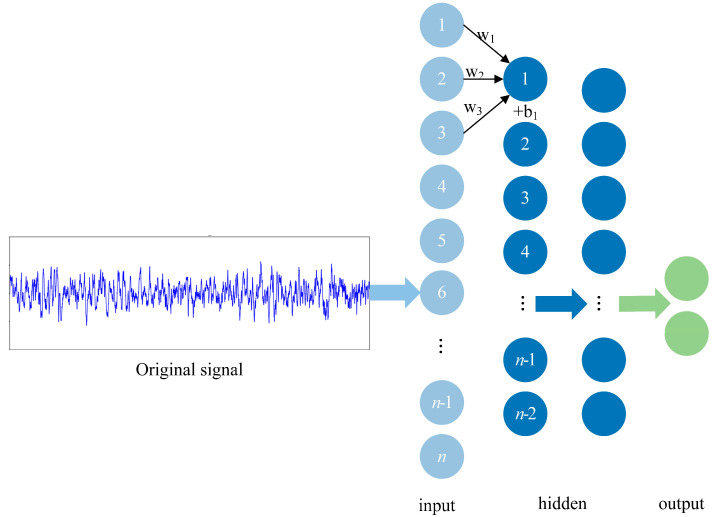
Schematic of 1D-CNN learning local features.

In this approach, we performed end-to-end epileptogenic signal identification on the original signal after processing. Figure 5 depicts the overall structure of 1D-CNN. Our study first selected a local signal frame and used this local signal frame to scan the entire signal.

1D-CNNs, first proposed by [33], involve nature language processing (NLP), which takes inputs of varying lengths and produces fixed-length vectors as output. Moreover, a 1D-CNN has many dimensional networks, few parameters, fast training speed, and an excellent overall effect. However, iEEG signals and NLP share the features of continuity and nonstationarity.

Thus, our study exploits the ability of a 1D-CNN to automatically detect and extract relevant features that may be too complex or subtle to be noticed by humans. The architecture we propose has four convolutional layers and two MLP layers. Each convolutional layer consists of a convolutional layer, a batch normalization layer, an activation layer and the maximum pooling layer. The specific structure interpretation and learning process of iEEG local features are shown in Figure 5.

**Figure 6:**
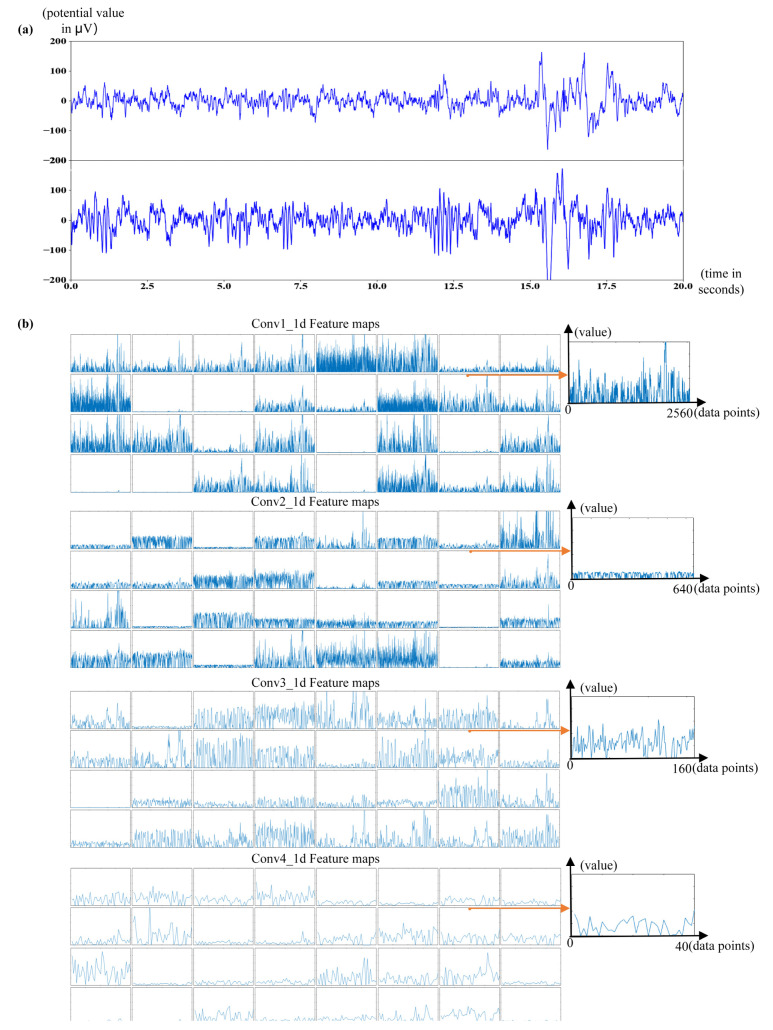
Original iEEG signals and feature maps from 1D-CNNs. (**a**)Schematic diagram of the original ES;(**b**) This is the Conv1-4_1d feature maps from 1D-CNNs, which represent signals learned by the detector in different convolutional layers. Specifically, since the 1D-CNNs model contains 32 channels, each layer contains 32 feature maps. Among them, the x-axis of each feature map represents the number of data points in the model learning and downsample process, and the *y*-axis represents the value of the data points. The right side of each layer has its corresponding feature map.

At the convolutional layer of the end-to-end model, convolution, multiple filters with different window sizes move on the iEEG data serials to perform one-dimensional convolution. As the filter moves on, many feature data, which capture the local correlation before and after the signal and minor changes, are generated. However, the data points of a signal have the most influence on the data points before and after it and have no relationship with data points farther from this data point. Thus, each neuron must be locally connected only to the previous layer, which is equivalent to scanning a small area around each neuron. Sharing the weights of many neurons is equivalent to scanning the global area, thus forming a feature map, as shown in Figure 6.

In the pooling layer, a max-overtime pooling operation is applied to capture the most useful local features from feature maps. Moreover, it can compress the amount of data and parameters and reduce overfitting. It has no parameters; it just downsamples the results given to it by the upper layer. Activation functions are added to increase the nonlinear expression ability of the model. The outputs of multiple filters are concatenated in the merge layer. After another dropout process, a fully connected SoftMax layer outputs the probability distribution over labels from multiple classes. At the fully connected layer of the end-to-end model, the previous local features are reassembled into a complete signal through the weight matrix. All neurons in the fully connected layer must be connected by weight at the end of 1D-CNN.

One dimensional convolutional neural networks have fewer parameters and faster training speed than 2D- or 3D-CNN. There are a few remarkable things to note in Figure 6b. The first layer acts as a local signal detector. At that stage, the activations retain almost all the information present in the initial signal, as shown in Figure 6b (conv1_1d). The features extracted by a layer become increasingly abstract with increasing depth of the layer and less visually interpretable. Moreover, the sparsity of the activations increases with increasing depth of the layer: in the first layer, all filters are activated by the input signal, but in the following layers, increasingly many data points are blank. They start encoding higher-level concepts, such as high-frequency oscillation or fast activity. Therefore, the activation of higher layers carries decreasing information about the specific input and increasing information about the class of the signal: epileptogenic or non-epileptogenic. Above all, a 1D-CNN effectively acts as an information distillation pipeline, with raw signal data entering and being repeatedly transformed so that irrelevant information is filtered out while useful information is magnified and refined.

### 2.6. Feature Extraction from Classical & DL Approach and Feature Fusion

**Figure 7:**
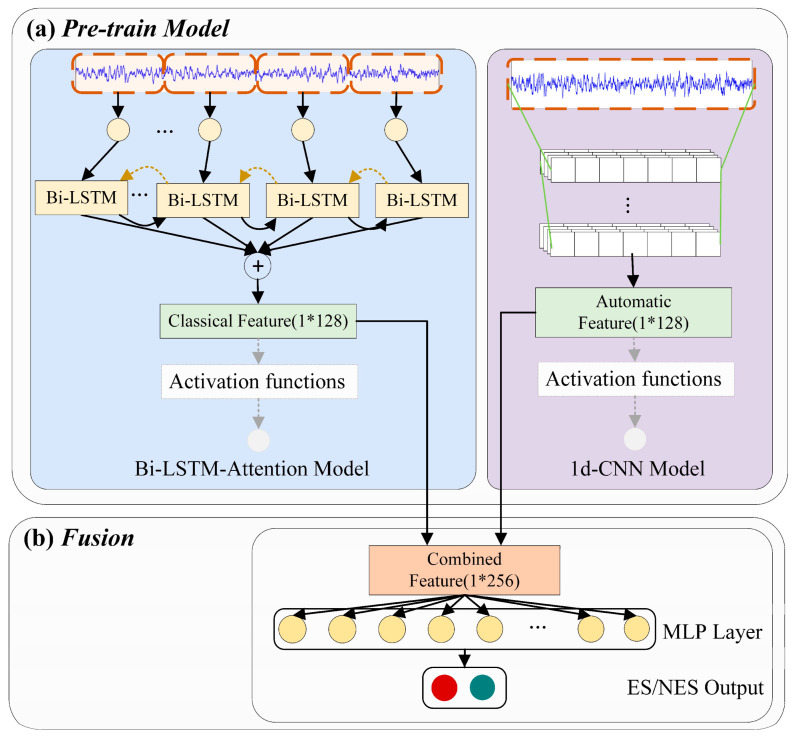
Overview of the multi-branch DL fusion model. (**a**) combine the signal feature from the classical approach and the automatic deep feature from the DL approach; (**b**) splice features into a fusion feature, then fed into an MLP neural network and mapped into two categories, ES and NES.

The core of our proposed method is the multi-branch DL fusion model, as shown in Figure 7.

We obtain features from the pretrained model, combine the signal feature from the classical approach and the automatic deep feature from the DL approach, and splice them end to end into a fusion feature. The specific dimensions of the classical feature are 1 × 128, and the automatic feature is 1 × 128; therefore, the dimension of the fusion feature is 1 × 256.

A binary cross-entropy loss function was employed for training as defined in Equation (7).

(7) $$l\left(y,\hat{y}\right)=-\left[y\log\left(\hat{y}\right)+\left(1-y\right)\log\left(1-\hat{y}\right)\right]$$

where $\hat{y}\;$and y are the desired output and the calculated output, respectively, and $l\left(y,\hat{y}\right)$ is the loss function.

Finally, the deep fusion feature is fed into an MLP neural network and mapped into two categories, ES and NES.

## 3. Results

### 3.1. Evaluation Metrics

The goal of our study is to judge whether iEEG originates from epileptogenic zones of the brain—that is, to achieve epileptogenic signal identification—which becomes a binary classification problem. Therefore, accuracy, sensitivity and specificity are essential indicators for evaluating the epileptogenic signal identification model.

The true positive count (TP) represents the number of epileptogenic signals correctly identified, the true negative count (TN) represents the number of non-epileptogenic signals correctly identified, the false positive count (FP) represents the number of signals falsely identified as epileptic signals, and the false negative count (FN) represents the number of signals falsely identified as non-epileptic signals.

Accuracy (ACC) evaluates the ratio of signals found and classified correctly as epileptogenic signals and non-epileptogenic signals by the model. Sensitivity (SE) and specificity (SP) evaluate the ratio of correctly found epileptogenic signals and non-epileptogenic signals, respectively. The specific formula is shown as follows as Equations (8)–(10):

(8) $$\mathrm{Accuracy}\left(\mathrm{ACC}\right)=\frac{TP+TN}{\left(TP+FP+TN+FN\right)}$$

(9) $$\mathrm{Sensitivity}\left(\mathrm{SE}\right)=\frac{TP}{\left(TP+FN\right)}$$

(10) $$\mathrm{Specificity}\left(SP\right)=\frac{TN}{\left(TN+FP\right)}$$

### 3.2. Parameter Setting

The optimal values for all these parameters in our study are presented in Table 3.

**Figure 8:**
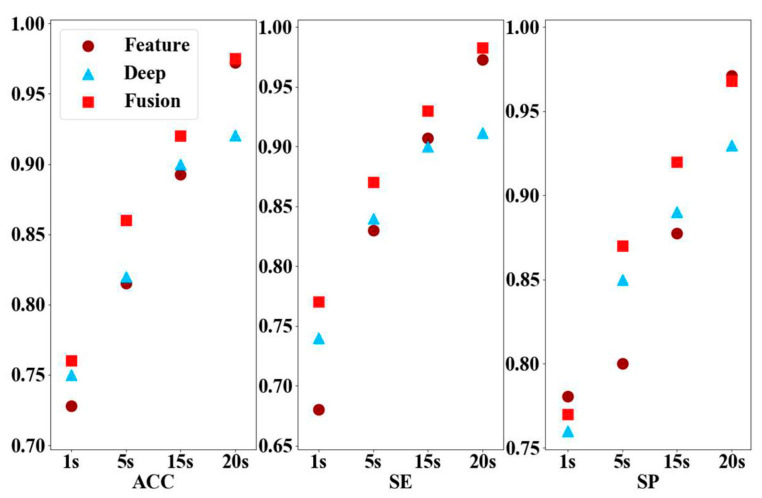
Changes in the size of the model input window according to the performance indices. The horizontal axis is the duration of the signal segment, in seconds. The vertical axis is the accuracy of different models.

To select the optimal input time window size for the epileptogenic signal identification model, our study experimented with different input time window sizes to evaluate the performance. According to the model network architecture, we can also accept 1 s input windows, 1, 5, 15, and 20 s, and the segmentation length of 20 s with 20,480 data points showed better performance because of its high temporal resolution. In this study, the segment size of public database was set to 20 s, and the private database contained 20,480 data points for feature extraction and signal identification. The performance indices with different input time windows are shown in Figure 8.

A number of parameters for the deep network were explored and tuned to achieve the best results for the deep fusion model, including the Bi-LSTM-Attention, 1D-CNN, and MLP classifiers. These hyperparameters included the LSTM hidden size, LSTM num layers, LSTM dropout, hidden linear size, linear dropout, batch size, and number of training epochs, which are applied after the Bi-LSTM-Attention input layer. Notably, the size of the kernel is an important hyperparameter of the 1D-CNN to tune. An MLP also contains hidden nonlinear layers. Additionally, some hyperparameters were tuned for the stochastic Adam optimizer [34].

### 3.3. Training and Testing Sets

For two public iEEG databases, we randomly selected 70% of all iEEG signals as training samples, and the remaining 30% were selected as the tested samples.

For the third private SEEG database, we prepared SEEG data of five cases for intra-subject and cross-subject training and testing. For intra-subject training and testing, in each subject, we randomly selected 60% of all samples as the training set of the proposed model, 10% as the validation set used to select accurate and stable model parameters, and the remaining 30% as the testing set used to judge the synthesis performance of the model. For cross-subject training and testing, using the leave-one-out method with a total of five subjects, four subjects’ data were randomly selected as the training set, and the remaining one subject’s data were used as the testing set.

### 3.4. Results Overview

Our study obtained iEEG data not only from two public benchmark iEEG databases but also from a private clinical SEEG database.

We achieved the epileptogenic signal identification model with high values of accuracy (97.60%), sensitivity (97.78%), and specificity (97.42%) in the Bern–Barcelona public database that contained 7500 signals of five patients, which is the highest performance in all experimental comparisons. Moreover, to select the optimal input time window size, our study experimented with different time window sizes to evaluate the performance. The segmentation length of 20 s showed better performance because of its high time resolution; even the segmentation length of 5 s can reach a sensitivity higher than 87.35%.

For the small public Born University database, we conducted experiments on subsets of its iEEG and achieved accuracy of 92.07%, sensitivity of 91.13%, and specificity of 92.96%.

Furthermore, our study validated the proposed model and achieved automatic epileptogenic signal identification on the clinical SEEG dataset that contained 64,890 signals of five patients. For the intra-subject experiment, we obtained an epileptogenic signal detection average accuracy of 92.53% (mean ± SE = 92.53% ± 0.0338), sensitivity of 93.18% (mean ± SE = 93.18% ± 0.0297), and specificity of 91.80% (mean ± SE = 91.80% ± 0.0395). For the cross-subject experiment, we achieved a performance evaluation index of increased accuracy (90.30%), sensitivity (89.06%), and specificity (91.58%). Our method was able to objectively identify the epileptogenic signals in five patients.

### 3.5. Evaluation Results Over Public iEEG Databases

#### 3.5.1. Results from the Public Bern–Barcelona Database

To illustrate the effectiveness of signal feature extraction and the advantage of the deep model to learn higher-order features automatically from the original iEEG signals, our study used a public iEEG database to train and verify the performance of the proposed deep fusion model. We use the Bern–Barcelona public dataset [21] to evaluate our epileptic iEEG signal identification model by public benchmarks.

The experiment extracted multiple features across multiple domains selected in the Methods section and compared them with different machine learning algorithms or single models, such as the support vector machine (SVM) [35], logistic regression (LR) [36], extreme randomized tree (ERT) [37], deep neural network (DNN), 1D-CNN and Bi-Stack-LSTM models [38]. The experimental results are shown in Table 4.

#### 3.5.2. Results from Small Public Bonn University Database

Few people use this dataset for iEEG epileptogenic signal identification. Our study compared several methods reproduced by ourselves, as shown in the Table 5. The evaluation metrics of the deep fusion model are much higher than in our previous research [15].

### 3.6. Evaluation Results over Private SEEG Database

#### 3.6.1. Intra-Subject Experiments Results

In the clinical SEEG database, we used the multi-branch DL fusion model for epileptogenic signal detection. In the experimental results, five cases with intractable epilepsy were put into the proposed model, and the average intra-subject accuracy was 92.53% (87.35–97.14%), as shown in Table 6.

#### 3.6.2. Cross-Subject Experimental Results

Our study used the leave-one-out method, and five sets of experiments were carried out. The average accuracy was 88.03% (85.63–90.30%), as shown in Table 7.

## 4. Discussion

The high-precision detection capabilities of the multi-branch DL fusion model of our study were illustrated not only on a public iEEG dataset but also on a clinical SEEG dataset.

### 4.1. Comparison with Other Methods

Our multi-branch DL fusion model can detect epileptogenic iEEG signal with high accuracy because of the exceptional feature extraction that combines DL features with multiple features across multiple domains. The multi-branch DL fusion model surpassed the performance of existing state-of-the-art techniques with accuracy of 97.60%, as shown in Table 8. We have proved that our multi-branch DL fusion model is effective on the Bern–Barcelona public dataset.

### 4.2. Multi-Branch Feature Extraction

The key of epileptogenic iEEG signal detection was to extract effective features to fully represent the signal. Our multi-branch DL fusion model used two approaches, the classical approach and the DL approach, which are completely different methods and ideas for signal processing.

**Figure 9:**
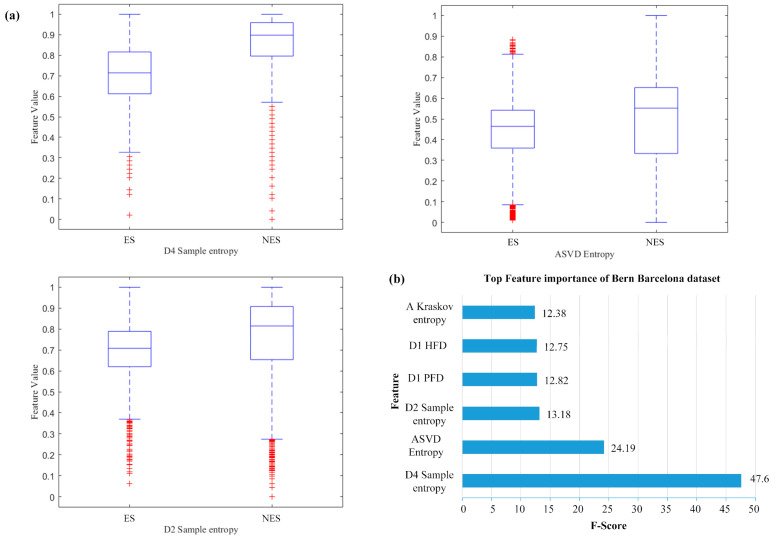
Feature analyses overview in our study using XGBoost, which shows the three most important features in the public dataset. (**a**) the top three most important features ranked, sample entropy of D4, SVD entropy of A4, and sample entropy of D2; (**b**) The top 5 features’ bar graph.

For the classical approach, Bi-LSTM-AM achieved better performance of accuracy (97.20%), sensitivity (97.29%), and specificity (97.10%) in the Bern–Barcelona dataset because of not only the extraction of multiple features but also the timing characteristics of the deep structures. We analyzed the distribution and contribution of the different features used in this study and calculated the top three most important features ranked, using XGBoost [41,42]: sample entropy of D4, SVD entropy of A4, and sample entropy of D2, where the difference between the means of the two classes (epileptogenic/non-epileptogenic) can be observed. Experiments showed that the three most important features in the data distribution can clearly distinguish epileptogenic signals from non-epileptogenic signals, as shown in Figure 9.

Moreover, we also analyzed features importance by clinical SEEG dataset, and the three top importance features ranked was HFD of D4, Hurst, and Kraskov entropy of D1. Furthermore, we analyzed the Bi-LSTM-Attention classifier of the classical approach. Its feature extraction ability is improved by introducing an AM and segmenting sequences into time segments. The Bi-LSTM-Attention classifier not only learns its features on the basis of a full signal but also learns time-dependent features, which can effectively detect small changes in iEEG.

For the DL approach, a 1D-CNN model is constructed for end-to-end identification of original signals and obtaining deep high-order features automatically, which obtained an epileptogenic signals detection accuracy of 89.87%. The activation of higher layers carries decreasing information about the specific input and increasing information about the class of the signal, deep higher-level concepts, such as high-frequency oscillation or fast activity, as shown in Figure 6.

Furthermore, we combined the classical approach and the DL approach as a pretrained model and obtained a fusion feature.

In summary, we proposed a multi-branch deep fusion model based on the classical approach and DL approach in which the combined features include not only basic signal features and time–frequency domain features in signal processing but also the time dependence of the signal, and further integrate the deep high-level features of the original signal. Therefore, the detection of epileptic signals benefits from high precision and low false positives from perfect multi-domain, multi-feature extraction and deep features extraction. It also provides a solid technical foundation for identifying the epilepsy origin of the signal.

### 4.3. Epileptogenic Signal Classification

The signal features selected in our study can effectively represent unstable, nonlinear iEEG signals. Moreover, the results of selecting the DL model as the classifier are superior to those of the machine-learning algorithm, indicating that when the feature data volume is larger, the deep structure can better fit high-dimensional data than traditional machine learning algorithms.

Our study also compares the training/testing times of the multi-branch DL fusion model. We noticeably decreased the testing time of the fusion model to 40 ms per signal as shown in Table 9. The DL approach automatically extracts higher-order features and realizes the identification of epileptic signals in a much shorter time than the classical approach. However, the sensitivity and accuracy of the end-to-end identification model differ from those of the classical approach by approximately seven percentage points. Although accuracy and test time are indicators that must be weighed, for iEEG-based epilepsy signal detection tasks, the accuracy rate has a higher priority than the test time.

### 4.4. Significance of Epileptogenic Signal Identification for the Localization of the Epileptic Zone

Only when a pathologically abnormal iEEG signal is detected can an abnormal lead be traced back and the localization of the epileptic zone be determined. Based on clinical studies, Liu et al. [43] considered high-frequency oscillations a reliable biomarker for seizure onset zone identification, and Patrick Chauvel’s team [44,45] used signal patterns, such as fast activity, low-frequency suppression, and preictal spikes, as fingerprints for epileptogenic zone positioning [11,13,46].

The experimental design and research ideas for the next step of this paper are to trace back the epileptogenic signal and use statistical features to locate it. After detection of the epileptic signal, our study traced it back to the epileptic lead contact. For example, one lead contact is labeled as the origin point. If 85% of the signals in the verification set where the contact is located are detected as epileptic signals, then the lead is an epileptic lead contact. Then, the epileptic lead contact to the MRI image is mapped, and its resection area label is verified. There is no reasonable logic to truly realize epileptogenic zone localization with digital signal characteristics. Therefore, there is scope for further research in this aspect.

### 4.5. Limitations of the Study

Due to the imbalance of positive and negative samples in clinical applications, our clinical SEEG dataset adopts a resampling method to balance the number of epileptic signals. The structure of our model does not solve the problem of data imbalance in practical applications.

In the cross-subject process, we assume that different kinds of epilepsy signals have the same imaging degree, and there is no specific disease type. However, it is influential to the clearer signal recognition of the lesion location. It also proves that our method is extremely robust.

## 5. Conclusions and Future Works

### 5.1. Conclusions

A multi-branch DL fusion model is proposed for the identification of epileptogenic signals from the epileptogenic area of the brain. Not only signal wave features but also deep high-order features are considered. Both branches use the DL model (Bi-LSTM-AM and 1D-CNN) as the classifier to high-level abstract the epileptogenic signal features based on a time-series feature sequence and raw time-series signal, and then two-class signals are accurately identified. Moreover, resampling is employed to split the imbalanced epileptogenic and non-epileptogenic samples into balanced subsets for clinical validation. This paper achieved not only state-of-the-art performance in the databases of two public evaluation benchmarks but also good application in a real-world clinical database. The proposed method is extremely robust while ensuring accuracy.

### 5.2. Future Work

Our work will focus on two aspects. On the one hand, we will focus on models capable of online epileptogenic signal detection rather than using an offline pretraining model.

On the other hand, localization of the epileptogenic zone is the ultimate goal of our follow-up research. In follow-up research, we will continue to collect a large amount of data on different types of epilepsy and focus on building more accurate models for the same type of epilepsy. From the perspective of improving the accuracy of epileptic signal detection, we will investigate the epileptic foci of specific disease identification. Combined with the different models that we have constructed, we can analyze the reasons for the differences in the performance of different diseases—that is, the significant difference characteristics and biomarkers. Moreover, the connection between signal patterns and digital features will be explored.

## Figures and Tables

**Table 1:** Summary of the clinical SEEG data in our study.

Subject ID	Sex F: Female M: Male	Age (Years)	Epilepsy Duration (Years)	Surgical Pathology	Proportion of ELs to NELs	Number of ES and NES
**Pt1**	M	21	5	FCD	4:69	2596:2622
**Pt2**	M	21	7	HS	10:66	10350:10362
**Pt3**	F	12	7	HH	6:56	8640:8680
**Pt4**	M	29	21	FCD	4:71	2880:2928
**Pt5**	F	8	4	FCD	11:23	7920:7912
**Total**	-	-	-	-	-	64890

Note: ELs: Epileptogenic leads, NELs: Non-epileptogenic leads, ES: Epileptogenic signals, NES: Non-epileptogenic signals, FCD: Focal cortical dysplasia, HS: Hippocampal sclerosis, HH: Hamartoma.

**Table 2:** Detailed characteristics and formulas of feature.

Category	Num	Name	Formula
Time domain	1	Mean	$\mu =\frac{1}{N}\overset{N}{\underset{i=1}{\sum }}x_{i}$
	2	Variance	$\sigma^{2}=\frac{1}{N}\overset{N}{\underset{i=1}{\sum }}{\left(x_{i}-\mu \right)}^{2}$
	3	Coefficient of variation	$cv=\frac{\left|\sigma \right|}{\mu }$
	4	Skewness	$s=\frac{\frac{1}{N}\sum_{i=1}^{N}{\left(x_{i}-\mu \right)}^{3}}{{\left(\frac{1}{N-1}\sum_{i=1}^{N}{\left(x_{i}-\mu \right)}^{2}\right)}^{3/2}}$
	5	Kurtosis	$k=\frac{\frac{1}{N}\sum_{i=1}^{N}{\left(x_{i}-\mu \right)}^{4}}{{\left(\frac{1}{N}\sum_{i=1}^{N}{\left(x_{i}-\mu \right)}^{2}\right)}^{2}}-3$
	6	IQR	$iqr=x_{sort}{}_{\left(N+1\right)\times 0.75}-x_{sort}{}_{\left(N+1\right)\times 0.25}$
	7	Activity	$act=\sigma^{2}$
	8	Mobility	$mob=\sqrt{\frac{\sigma_{x^{\prime }}{}^{2}}{\sigma^{2}}}$
	9	Complexity	$com=\sqrt{\frac{\sigma_{x^{\prime \prime }}{}^{2}/\sigma_{x^{\prime }}{}^{2}}{\sigma_{x^{\prime }}{}^{2}/\sigma^{2}}}$
	10	Zero-crossing	$ZC=\frac{1}{2}\overset{N}{\underset{i=1}{\sum }}\left|sgn\left(x_{i}\right)-sgn\left(x_{i-1}\right)\right|$
	11	Hurst	$H=\log_{N}\left(C\cdot E\left[\frac{\begin{array}{c} max\left(L_{1},L_{2},\cdots ,L_{N}\right)\\ -min\left(L_{1},L_{2},\cdots ,L_{N}\right) \end{array}}{\left|\sigma \right|}\right]\right),$ $L_{i}=\sum_{i=1}^{N}(x_{i}-\mu )$
	12	DFA	$F\left(n\right)=\sqrt{\frac{1}{N}\overset{N}{\underset{i=1}{\sum }}{\left(\overset{N}{\underset{i=1}{\sum }}(x_{i}-\mu )-y_{n}\left(i\right)\right)}^{2}}\propto n^{\alpha }$
Frequency domain	13	Sub-band power ratio (60–140 Hz/0–60 Hz)	$SPR=\frac{\int_{\omega_{1}}^{\omega_{2}}s_{xx}\left(\omega \right)d\omega }{\int_{\omega_{2}}^{\omega_{3}}s_{xx}\left(\omega \right)d\omega }$
	14	Power spectral density	$s_{xx}\left(\omega \right)=\underset{T\to \infty }{\lim}E\left[{\left|\hat{X}\left(\omega \right)\right|}^{2}\right]$
	15	Amplitude spectral density	$\hat{X}\left(\omega \right)=\frac{1}{\sqrt{T}}\int_{0}^{T}x\left(t\right)exp^{-i\omega t}dt$
	16	Spectral centroid	$SC=\frac{\sum_{i=1}^{N}f_{i}\cdot p\left(f_{i}\right)}{\sum_{i=1}^{N}p\left(f_{i}\right)}$
	17	Spectral kurtosis	$SK=\frac{\sum_{i=1}^{N}{\left(f_{i}-sc\right)}^{4}p\left(f_{i}\right)}{sp^{4}\sum_{i=1}^{N}p\left(f_{i}\right)}$
	18	Spectral entropy	$SE=\frac{-\sum_{i=1}^{N}P_{i}\cdot \log P_{i}}{\log\left(length\left(P_{i}\right)\right)},P=\frac{x_{i}}{\sum_{i=1}^{N}x_{i}}$
TF domain	19–20	Fuzzy entropy	$FE=\ln O^{m}\left(m,r\right)-\ln O^{m+1}\left(m,r\right)$
Non-linear	21–25	Kraskov entropy	$\hat{KE}\left(X\right)=-\varphi \left(k\right)+\varphi \left(N\right)+\log\left(V_{d}\right)$ $+\cdots \frac{d}{N}\overset{N}{\underset{i=1}{\sum }}\log\left(2\delta \left(x_{i},k\right)\right),$ $V_{d}=\pi^{d/2}/\Gamma \left(1+d/2\right)/2^{d},k=4$
	26–30	Renyi entropy	$RE_{\alpha }\left(X\right)=\frac{1}{1-\alpha }\log\left(\overset{N}{\underset{i=1}{\sum }}p_{i}^{\alpha }\right),\alpha =2$
	31–35	Permutation entropy	$PE_{D}=-\frac{1}{\log_{2}D!}\overset{D!}{\underset{i=0}{\sum }}p_{i}\log_{2}p_{i},D=3$
	36–40	Sample entropy	$SaE=-\ln\left[\frac{{\left(N-k+1\right)}^{-1}\sum_{i=1}^{N-k+1}A_{i}^{k}\left(r\right)}{{\left(N-m+1\right)}^{-1}\sum_{i=1}^{N-m+1}B_{i}^{m}\left(r\right)}\right],$ $m=2,r=0.2\times \left|\sigma \right|,k=m+1$
	41–45	Shannon entropy	$ShE\left(X\right)=\underset{\alpha \to 1}{\lim}RE_{\alpha }\left(X\right)=-\overset{N}{\underset{i=1}{\sum }}p_{i}\log p_{i}$
	46–50	Energy	$E=\overset{N}{\underset{i=1}{\sum }}x_{i}{}^{2}$
	51–55	SVD Entropy	$Svd=-\frac{1}{\log\left(N\right)}\overset{N}{\underset{j=1}{\sum }}\left(\frac{s_{j}^{2}}{\sum_{k}s_{k}^{2}}\right)\log\left(\frac{s_{j}^{2}}{\sum_{k}s_{k}^{2}}\right)$
	56–60	PFD	$P_{D}=\frac{\log n}{\log n+\log\left(\frac{n}{n+0.4N}\right)}$
	61–65	KFD	$K_{D}=\frac{\log n}{\log\left(\frac{d}{L}\right)+\log n}$
	66–70	HFD	$H_{D}=\ln\left(\overset{k}{\underset{m=1}{\sum }}L_{m}\left(k\right)\right)$

Note: TF: Time and frequency, IQR: Inter quartile range, DFA: Detrended fluctuation analysis, SVD: Singular value decomposition, PFD: Petrosian fractal dimension, KFD: Katz fractal dimension, HFD: Higuchi fractal dimension.

**Table 3:** Training parameters.

Parameters	Bi-LSTM-Attention	Parameters	1D-CNN
LSTM hidden size	64	Conv num layers	4
LSTM num layers	2	(in, out, kernel size, stride, padding) of layer1	(1,16,3,1,1)
LSTM dropout	0.1	(in, …, padding) of layer2	(16,32,3,1,1)
hidden linear size	256	(in, …, padding) of layer3	(32,32,3,1,1)
linear dropout	0.3	(in, …, padding) of layer4	(32,32,2,1,1)
batch size	20	batch size	32
training epochs	100	training epochs	100

**Table 4:** Results from the multi-branch DL fusion model with public Bern–Barcelona dataset.

Method	Extracted Feature	ACC	SE	SP
SVM	70 various features	90.87%	-	-
LR	70 various features	92.27%	-	-
ERT	70 various features	92.67%	-	-
1D-CNN	70 various features	95.33%	95.06%	95.62%
DNN	70 various features	96.80%	96.88%	96.72%
LSTM	70 various features × 4 segments	94.80%	96.48%	93.18%
Bi-LSTM	70 various features × 4 segments	95.13%	95.84%	94.43%
Bi-LSTM-AM	70 various features × 4 segments	97.20%	97.29%	97.10%
DNN	Automatic	58.07%	55.74%	60.61%
Stack LSTM	Embedding + Automatic	87.87%	88.13%	87.59%
1D-CNN	Automatic	89.87%	90.13%	89.59%
Proposed	70 various features × 4 segments + Automatic	97.60%	97.78%	97.42%

**Table 5:** Results from the multi-branch DL fusion model with small public Bonn University database.

Method	Extracted Feature	ACC	SE	SP
DNN	70 various features	82.50%	84.21%	80.95%
Bi-LSTM-AM	70 various features × 4 segments	85.00%	89.47%	80.95%
1D-CNN	Automatic	90.00%	94.74%	85.71%
Proposed	70 various features × 4 segments + Automatic	92.07%	91.13%	92.96%

**Table 6:** Results from the multi-branch DL fusion model with clinical SEEG dataset using intra-subject scheme (patients 1–5).

Patients	Method	Extracted Feature	ACC	SE	SP
Pt1	Bi-LSTM-AM	70 various features × 4 segments	95.31%	96.18%	94.35%
	1D-CNN	Automatic	95.59%	94.96%	96.31%
	Proposed	70 various features × 4 segments + Automatic	97.14%	96.85%	97.44%
Pt2	Bi-LSTM-AM	70 various features × 4 segments	87.50%	80.95%	94.74%
	1D-CNN	Automatic	86.75%	92.51%	91.35%
	Proposed	70 various features × 4 segments + Automatic	87.35%	88.47%	85.82%
Pt3	Bi-LSTM-AM	70 various features × 4 segments	93.91%	93.18%	94.64%
	1D-CNN	Automatic	84.93%	82.49%	87.55%
	Proposed	70 various features × 4 segments + Automatic	91.09%	92.09%	89.96%
Pt4	Bi-LSTM-AM	70 various features × 4 segments	90.93%	91.37%	90.46%
	1D-CNN	Automatic	89.59%	90.75%	88.34%
	Proposed	70 various features × 4 segments + Automatic	91.96%	92.63%	91.40%
Pt5	Bi-LSTM-AM	70 various features × 4 segments	94.20%	93.33%	95.14%
	CNN	Automatic	91.38%	91.86%	90.86%
	Proposed	70 various features × 4 segments + Automatic	95.13%	95.84%	94.43%

**Table 7:** The results for the multi-branch DL fusion model with the clinical SEEG dataset using the cross-subject scheme (patients 1–5).

Patients Set	Method	Extracted Feature	ACC	SE	SP
Pt1-4/Pt5	Bi-LSTM-AM	70 various features × 4segments	83.28%	84.40%	82.18%
	1D-CNN	Automatic	77.68%	82.52%	72.65%
	Proposed	70 various features × 4 segments + Automatic	87.59%	88.58%	86.62%
Pt2-5/Pt1	Bi-LSTM-AM	70 various features × 4 segments	85.16%	86.32%	84.03%
	1D-CNN	Automatic	78.42%	82.73%	74.21%
	Proposed	70 various features × 4 segments + Automatic	87.68%	87.16%	88.21%
Pt3-5,1/Pt2	Bi-LSTM-AM	70 various features × 4 segments	88.59%	82.96%	91.92%
	1D-CNN	Automatic	85.11%	86.30%	83.89%
	Proposed	70 various features × 4 segments + Automatic	90.30%	89.06%	91.58%
Pt4-5,1-2/Pt3	Bi-LSTM-AM	70 various features × 4 segments	86.20%	83.08%	89.41%
	1D-CNN	Automatic	78.14%	86.87%	69.14%
	Proposed	70 various features × 4 segments + Automatic	88.93%	88.42%	89.41%
Pt5,1-3/Pt4	Bi-LSTM-AM	70 various features × 4 segments	83.82%	86.09%	81.63%
	CNN	Automatic	80.59%	83.97%	77.16%
	Proposed	70 various features × 4 segments + Automatic	85.63%	87.91%	83.42%

**Table 8:** Comparison of the Bern–Barcelona public dataset DL fusion model experimental results.

Method/Year	Extracted Feature	Classifier	ACC	SE	SP
Das and Bhuiyan [39]	EMD-DWT, log energy entropy	KNN	89.40%	-	-
Sriraam and Raghu [40]	26 various features	SVM	92.15%	94.56%	89.74%
Rahman et al. [18]	VMD-DWT	Ensemble stacking	96.1%	94.4%	95.2%
Li et al. [15]	Entropy-based features	RBF	93.91%	92.94%	94.88%
The Proposed (2021)	70 various features × 4 segments + Automatic feature	Proposed Model	97.60%	97.78%	97.42%

**Table 9:** Comparison of training/testing time of the epileptogenic signal identification method. We calculate the training and testing times of the single methods and deep fusion model separately, using three iEEG databases.

Method	Manual Feature (ms)			Train (ms)			Test (ms)		
	1	2	3	1	2	3	1	2	3
Bi-LSTM-AM	18874	7549	37723	2	1	5	1	1	2
1D-CNN	-	-	-	47	19	93	40	16	81
Proposed Model	18874	7549	37723	50	20	98	41	17	83

## Data Availability

Demo data and code resource libraries can be inquired, by email to the authors.
